# Spreading Degree Modulates Floral Aroma Development in Green Tea: Integrated GC-E-Nose, Metabolomics, and Molecular Docking Reveals Key Odorants and Olfactory Receptor Interactions

**DOI:** 10.3390/foods15040735

**Published:** 2026-02-16

**Authors:** Jiajing Hu, Xianxiu Zhou, Guangyue Hou, Jiahao Tang, Yongwen Jiang, Haibo Yuan, Daliang Shi, Yanqin Yang

**Affiliations:** 1 National Key Laboratory for Tea Plant Germplasm Innovation and Resource Utilization, Tea Research Institute, Chinese Academy of Agricultural Sciences, Hangzhou 310008, China; 2 College of Tea Science, Yunnan Agricultural University, Kunming 650201, China; 3 Shandong Institute for Product Quality Inspection, Jinan 250102, China; 4 Tea Research Institute, Hangzhou Academy of Agriculture, Hangzhou 310024, China

**Keywords:** green tea, spreading degree, floral aroma, GC-E-nose, targeted metabolomics

## Abstract

The spreading process constitutes a pivotal stage in green tea manufacturing. This study integrated GC-E-Nose with targeted metabolomics to comprehensively elucidate the dynamic changes in sensory characteristics and aroma substances of green tea across varying spreading degrees. Our findings demonstrated that spreading degree significantly modulated green tea’s aroma profile, with lighter degree particularly promoting the development of desirable floral aroma. GC-MS/MS quantification identified 70 volatile compounds, among which 38 exhibited spreading-dependent differential accumulation (VIP > 1.0, *p* < 0.05). Five key odorants, including indole, *β*-ionone, nerolidol, *cis*-jasmone, and *β*-damascenone, were highlighted as essential contributors to the floral aroma. Molecular docking simulations indicated stronger binding affinities between these five odorants and the olfactory receptor OR1D2 (<−6 kcal/mol), primarily via hydrogen bonding and hydrophobic interactions. These findings indicate that modulating the spreading degree is an effective processing strategy to enhance the development of floral aroma in green tea, offering valuable insights for precision-driven optimization of tea processing protocols.

## 1. Introduction

Green tea is highly valued for its delicate flavor, well-documented health benefits, and profound cultural significance [1,2]. Its distinctive sensory properties and broad biological functions of green tea are attributed to a complex array of bioactive components, primarily volatile compounds, amino acids, polyphenols, and alkaloids [3]. The flavor quality of green tea varies considerably due to differences in cultivars, growing environments, and processing techniques. Based on the methods of fixation and drying, green tea is commonly classified into four main types: pan-fired, baked, sun-dried, and steamed [4].

The advancement of processing technology is pivotal for enhancing the aroma quality of green tea. Spreading, the initial and critical step in green tea production, facilitates not only gradual dehydration but also a cascade of complex chemical transformations. As the process proceeds, cellular respiration declines, cells undergo shrinkage, and leaf texture softens [5]. Simultaneously, enzyme activity increases, promoting the oxidation and degradation of macromolecular substances such as proteins, polysaccharides, and lipids, thereby providing a robust material foundation for the development of flavor and aroma [6]. Previous studies have indicated that using warm-color light irradiation during spreading, especially yellow light, significantly improves green tea aroma quality [7]. However, the mechanistic relationship between differential spreading degrees and their modulation of green tea’s sensory properties and volatile compounds remains unresolved, thereby constraining technological innovation in process optimization.

Aroma constitutes a paramount quality indicator in tea evaluation, contributing approximately 25% to the total sensory assessment score and serving as a decisive factor in consumer preference. The aromatic complexity of tea arises from the synergistic interplay of numerous volatile compounds such as alcohols, aldehydes, ketones, and terpenoids, which collectively account for only 0.005~0.020% of tea dry matter [8,9]. Despite their trace concentrations, these compounds form the molecular foundation of tea aroma, with subtle compositional shifts dramatically altering sensory profiles [10]. Traditional aroma quality evaluation primarily depends on expert sensory assessment. However, prolonged evaluation and fatigue may affect the repeatability of sensory assessment, while the physiological and psychological states of evaluators can also impact the results. Intelligent sensory technologies such as gas chromatography-based electronic nose (GC-E-Nose) have emerged and are widely applied in flavor analysis, showing unparalleled effectiveness in quality assessment [11]. Metabolomics, a novel omics technology subsequent to proteomics, transcriptomics, and genomics, enables the enhancement of food flavor quality through the analysis of metabolite changes in response to food processing or stimulation [12,13,14]. In recent years, metabolomics has emerged as a powerful analytical platform for comprehensive profiling of flavor-related compounds during tea processing, leveraging its unparalleled capacity for simultaneous compound identification and quantitative analysis [15].

Odor perception begins with the binding of odorant molecules to olfactory receptors (ORs), a process that has garnered increasing research attention in recent years [16]. Broad-spectrum receptors such as OR1A1 and OR1D2 exhibit responsiveness to diverse aromatic ligands, making them pivotal models for studying aroma-receptor interaction mechanisms [17]. Molecular docking has emerged as a powerful computational strategy for visualizing and predicting atomic-level interactions between chemosensory receptors (olfactory/taste) and small molecules [18]. This approach allows for the efficient screening of flavor-active compounds and helps elucidate the mechanisms underlying flavor perception. Specifically, it advances our understanding of odor categories such as caramel-like, floral, and fresh aromas, while providing critical references for deciphering the molecular basis of characteristic fragrance formation [16].

In this study, the impact of different spreading degrees on green tea’s sensory quality and aroma compounds was comprehensively analyzed by integrating GC-E-Nose and targeted metabolomics. The key odorants that influenced the development of floral aroma formation were identified, and their interactions with olfactory receptors were characterized via molecular docking analysis. The results offer novel insights into spreading-degree-driven modulation of the floral aroma quality of green tea.

## 2. Materials and Methods

### 2.1. Materials and Reagents

Headspace vials (20 mL) sealed with 18 mm magnetic PTFE/silicone caps were obtained from Agilent Technologies Inc. (Palo Alto, CA, USA). Purified water, which was employed throughout the experimental procedures, was provided by Hangzhou Wahaha Group Co., Ltd. (Hangzhou, China). A detailed list of the standard substances is provided in Table S1.

### 2.2. Sample Preparation

The tea cultivar used in this study was “Longjing-changye”, characterized by a tenderness of one bud and one leaf. Fresh leaves with a moisture content of 76.09% were harvested in late April 2023 in Shengzhou City, Zhejiang Province. The green tea was processed according to previously methods, mainly including spreading (under yellow light irradiation), fixation, rolling, first drying, and final drying [7]. The specific processing parameters are shown in Figure S1. The environmental parameters during the spreading process were strictly controlled and continuously monitored using an artificial climatic chamber (PRX-4500, Ningbo Prant Instrument Co., Ltd., Ningbo, China). The tea samples were withered to moisture contents ranging from 73.36% (light withering) to 67.53% (over-withering), representing a gradient of withering intensity. They were correspondingly marked as S1, S2, S3, and S4. These four stages (S1–S4) represent progressive dehydration during spreading, a process that likely influences the leaf’s physiological state and enzyme-substrate interactions critical for volatile formation. All finished tea samples were stored at −20 °C prior to analysis. Three independent processing batches were conducted for reproducibility.

### 2.3. Sensory Evaluation

Sensory evaluation was conducted by a panel of six trained panelists (three females and three males), in accordance with GB/T 23776-2018 [19]. Prior to formal assessment, all panelists completed structured training to calibrate their sensory perception using a standardized aroma lexicon and representative tea samples. All assessors provided informed consent for participation and data utilization. Ethical approval was deemed unnecessary. Aroma quality was scored on a structured 100-point descriptive scale defined by the aforementioned standard, where higher scores indicate superior aroma quality. Specifically, the scale corresponds to official quality grades: scores of 90–99 represent the highest grade; scores of 80–89 represent good quality; scores of 70–79 represent acceptable quality. The evaluation procedure involved the precise measurement of 3 g tea samples combined with 150 mL of boiling water, which was then placed in a porcelain cup specifically designed for tea assessment. After a controlled brewing period of 4 min, the tea infusions were filtered into an evaluation bowl. Assessors independently rated aroma attributes and assigned scores to the aroma quality. Each sample was evaluated in triplicate across different sessions to ensure reproducibility.

### 2.4. GC-E-Nose Analysis

Volatile profiles of green tea samples with different spreading degrees were analyzed using a GC-E-Nose system (Alpha M.O.S., Toulouse, France). The experimental parameters were modified from previously established protocols [20]. Tea samples (0.5 g) were sealed in 20 mL headspace vials and incubated at 60 °C for 20 min with agitation (500 rpm), without the addition of water or any other substances. After incubation, 5000 µL of headspace gas was injected into the GC-E-Nose at a constant flow rate of 300 µL/s. Volatiles were trapped on Tenax TA at 20 °C for 27 s and then rapidly heated to 240 °C to release the trapped compounds into the GC system. Volatile separation was achieved using a parallel dual-column fast GC system equipped with MXT-5 and MXT-1701 columns (20 m × 0.18 mm I.D. × 0.4 μm; Restek, Bellefonte, PA, USA), which provided complementary separation owing to their differing polarities. The column temperature program started with a 5 s hold at 50 °C, increased to 80 °C at 0.1 °C/s, then ramped to 250 °C at 0.4 °C/s, and held at 250 °C for 10 s. The total run time was 740 s, and both FIDs were set to 260 °C for data acquisition.

### 2.5. GC-MS/MS Analysis

A modified HS-SPME technique, adapted from a previous study [21], was employed for sample extraction. For this process, tea samples (0.5 g) were homogenized with 5 mL of purified water in a headspace vial (20 mL). A DVB/CAR/PDMS fiber was subsequently exposed to the vial headspace, and the vial was incubated at 60 °C for 60 min without agitation. Following the incubation period, the fiber was thermally desorbed in the injection port (230 °C) for 5 min, allowing the release of adsorbed compounds into the chromatographic system for subsequent analysis.

Volatile profiling was conducted using an Agilent 7890A gas chromatograph coupled with an Agilent 7000C triple quadrupole mass spectrometer (Agilent Technologies, CA, USA) equipped with a DB-5MS column (30 m × 0.25 mm I.D. × 0.25 μm). The oven temperature program started at 40 °C (held for 5 min), increased to 160 °C at 4 °C/min, and was held at 160 °C for 5 min. The injector was operated in splitless mode, with helium as the carrier gas at 1.0 mL/min. The ion source and transfer line temperatures were set to 230 °C and 250 °C, respectively. The MS/MS analysis was performed in multiple reaction monitoring (MRM) mode, with MS1 operated at wide resolution and MS2 at unit resolution. High-purity nitrogen (99.999%) was used as the collision gas (1.5 mL/min), and helium was used as the quench gas (2.25 mL/min).

Chromatographic alignment and data preprocessing were performed using the MassHunter Quantitative Analysis software (version B.07.01, Agilent Technologies). Identification of volatile constituents was conducted through a systematic approach comprising two steps: (1) spectral matching of experimental data against the NIST 14 mass spectral library, and (2) confirming using authentic chemical standards. Following untargeted GC-MS/MS profiling of representative samples to select the target volatiles, absolute quantification was performed through an external standard calibration method, with detailed quantitative data provided in Table S2.

### 2.6. Odor Activity Value Analysis

The odor activity value (OAV) is a pivotal tool for bridging chemical composition and sensory perception in aroma studies. Compounds exhibiting OAV > 1 are typically classified as pivotal contributors, as their concentrations exceed human sensory detection thresholds, making them instrumental in shaping the perceived aroma. The OAV is defined by the ratio of a compound’s measured concentration (C) to its odor threshold (OT) in water, expressed mathematically as: OAV = C/OT.

### 2.7. Molecular Docking

The olfactory receptors OR1A1 (UniProt ID: Q9P1Q5), OR1D2 (UniProt ID: P34982), OR1G1 (Uniport ID: P47890), OR2W1 (UniProt ID: Q9Y3N9), and OR5M3 (UniProt ID: Q8NGP4), along with five characteristic aroma compounds (indole, *β*-ionone, nerolidol, *cis*-jasmone, and *β*-damascenone), were selected for interaction analysis. Three-dimensional structural models of ORs were obtained from the UniProt database (https://www.uniprot.org/, accessed on 22 May 2025), while ligand structures were acquired from PubChem (https://pubchem.ncbi.nlm.nih.gov/, accessed on 22 May 2025). Molecular docking simulations were conducted using AutoDock software Version 4.2 (Scripps Research, La Jolla, CA, USA, accessed on 22 May 2025,). The docking complex structures were obtained at Pymol (DeLano Scientific LLC, San Francisco, CA, USA, accessed on 22 May 2025) and visualized using AutoDock Tools Version 1.5.6 (Scripps Research, USA, accessed on 22 May 2025).

### 2.8. Statistical Analysis

All experimental replicates were performed in triplicate to ensure reproducibility and to minimize variability. The data were reported as mean ± standard deviation. Multivariate data patterns were analyzed using OPLS-DA in SIMCA 14.0 (Umetrics, Umeå, Sweden). Differences in aroma scores and compound concentrations among spreading-degree groups were compared by one-way ANOVA with Tukey’s post hoc test (*p* < 0.05) in SPSS 20.0 (IBM Corporation, New York, NY, USA). Hierarchical cluster analysis (HCA) was conducted by the Metware Cloud platform (https://cloud.metware.cn, accessed on 19 May 2025).

## 3. Results and Discussion

### 3.1. Analysis of Aroma Quality Based on Sensory Evaluation

The four spreading degrees examined in this study were selected to reflect common industrial practice, representing a practical moisture gradient in green tea processing. As summarized in Table S3, different spreading degrees exerted a marked impact on the aroma profiles. Notably, the S1 treatment exhibited distinct floral dominance and achieved the highest aroma score (90.83 points on a 100-point scale), indicating that lighter spreading optimally preserves or enhances floral notes. S2 was characterized by a subtle floral note, suggesting intermediate spreading may partially retain floral compounds but with less intensity than S1. S4 and S3 exhibited a pure and fragrant aroma, possibly due to biochemical changes (e.g., oxidation or volatilization) that reduce floral intensity, resulting in simpler aromatic profiles. Collectively, these results indicated that the spreading degree directly governs the sensory quality of green tea. The superior floral aroma in S1 is likely attributable to the better preservation of key volatile organic compounds, possibly through minimized oxidative degradation or maintained activity of aroma-forming enzymes.

### 3.2. Effect of Spreading Degree on the Volatile Fingerprints Analyzed by GC-E-Nose

To complement conventional sensory evaluation, the volatile fingerprints of teas processed under different spreading degrees were analyzed using a GC-E-Nose. This intelligent sensory technology employs dual gas chromatographic columns of distinct polarities for high-throughput separation.

To gain insight into the dynamic alterations of volatile fingerprints, a supervised OPLS-DA model was applied for analysis. This multivariate method employs partial least squares regression to construct predictive models correlating volatile metabolite expressions with sample classifications. As illustrated in Figure S2A, the OPLS-DA score plot demonstrated pronounced separation among the four spreading treatments, confirming distinct differences in aroma fingerprint profiles. The parameters of the model exhibited a high degree of reliability, as evidenced by an explanatory power for the dependent variable (R^2^Y) of 0.993 and a predictive power (Q^2^) of 0.557. Furthermore, the model’s robustness was additionally evaluated using a permutation test consisting of 200 iterations, which produced favorable parameters (R^2^ = 0.745 and Q^2^ = −0.609) as illustrated in Figure S2B. Overall, the GC-E-Nose analysis not only corroborated the sensory evaluation results but also demonstrated its efficacy in rapid discrimination of teas subjected to differential spreading degrees.

### 3.3. Different Spreading Degrees Modulate the Volatile Components Analyzed via GC-MS/MS

#### 3.3.1. Comparison of Aroma Components in Green Tea Across Different Spreading Degrees

**Figure 1:**
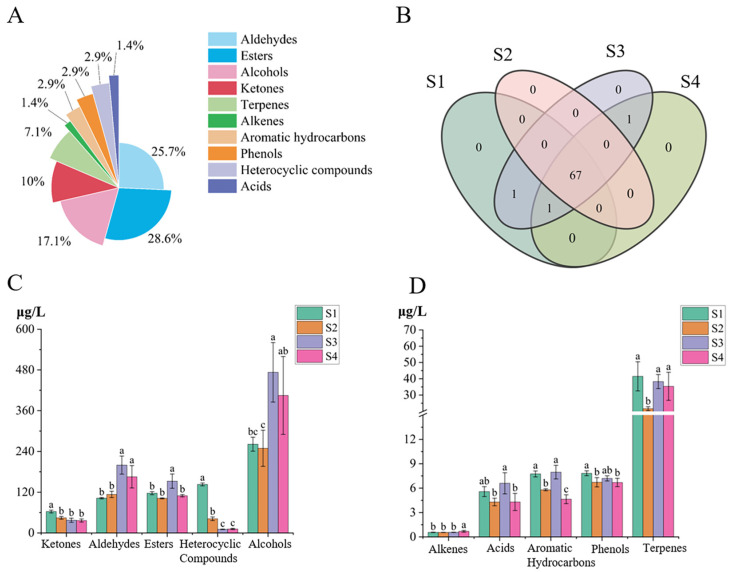
The information on volatile compounds in green tea samples with different spreading degrees analyzed by GC-MS/MS. (**A**) Proportion of identified volatile compounds by chemical class; (**B**) Venn diagram of volatile compounds; (**C**,**D**) Content comparison of different categories of volatile compounds. Statistical differences were indicated by different letters (*p* < 0.05). S1, S2, S3 and S4 represent the moisture contents of 73.36%, 71.40%, 69.17%, and 67.53%, respectively.

Targeted GC-MS/MS analysis was conducted to accurately quantify the volatile components in green tea processed under various spreading degrees. Totally, 70 volatile components were identified and categorized into ten distinct chemical classes: 20 esters, 18 aldehydes, 12 alcohols, 2 heterocyclic compounds, 7 ketones, 1 alkene, 2 phenols, 5 terpenes, 1 acid, and 2 aromatic hydrocarbons (Table S4). Among them, esters (28.6%), aldehydes (25.7%), and alcohols (17.1%) accounted for the largest proportions of the identified volatile compounds (depicted in Figure 1A). Notably, 67 volatile components were consistently detected across the four spreading degrees, while three treatment-specific markers exhibited distinct distribution patterns (in Figure 1B). For instance, *(E)*-2-hexenal was detected only in treatments S3 and S4; 3-Octanone was present in three treatments, excluding S2; *β*-Cyclocitral was detected solely in treatments S1 and S3.

The distribution of different categories of volatile compounds under varying spreading treatments is presented in Figure 1C,D, with alcohols emerging as the most abundant aroma-contributing components. Notably, the content of alcohols reached its highest level in S3 (473.02 μg/L), demonstrating significant elevations compared to S1 and S2 (*p* < 0.05). Aldehydes, key contributors to green tea aroma, showed higher contents in S3 and S4 than in S1 and S2. Among them, phenylacetaldehyde was the most prevalent aldehyde, with the maximal level of 115.46 μg/L in S3 and the minimal of 63.00 μg/L in S2. Esters attained their highest concentration in S3 (152.26 μg/L), surpassing the levels in other treatments. Heterocyclic compounds, associated with caramel-like and roasted notes, displayed pronounced variability, decreasing from 142.91 μg/L in S1 to 10.88 μg/L in S3. Ketones were most abundant in S1, and terpene levels ranked as S1 > S3 > S4 > S2. Among other quantified volatiles, acids and aromatic hydrocarbons peaked in S3, whereas phenols were highest in S1. These findings demonstrate that spreading degrees significantly altered the volatile compositions of green tea, with ketones and heterocyclic compounds exhibiting progressive declines as the spreading degrees increase (Figure 1C).

#### 3.3.2. Multivariate Statistical Analysis of the Volatile Compounds

**Figure 2:**
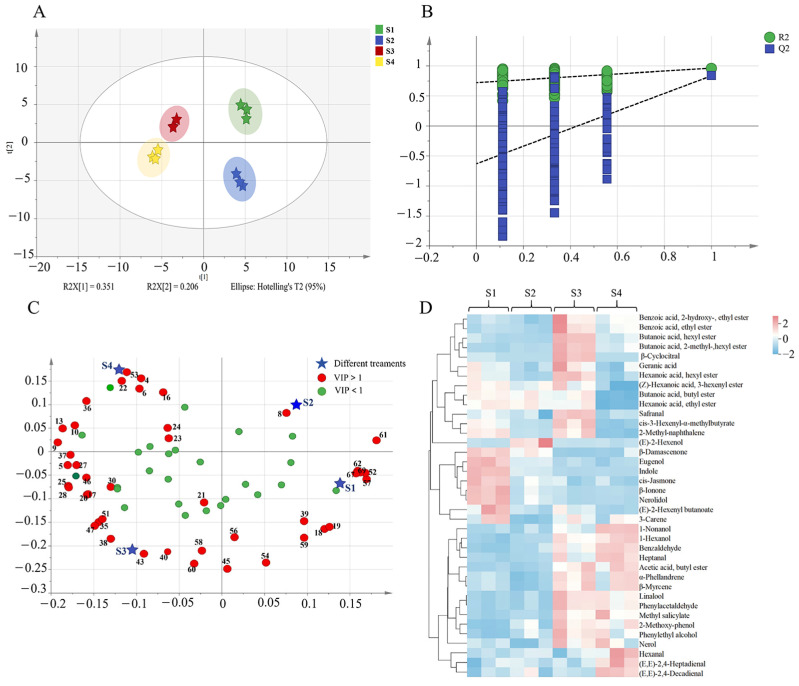
Multivariate statistical analysis on the volatile compounds obtained from GC-MS/MS. (**A**) The score plots of OPLS-DA (R^2^Y = 0.957, Q^2^ = 0.805); (**B**) Cross-validation by a 200-times permutation test (R^2^ = 0.737, Q^2^ = −0.607); (**C**) Loading plot; (**D**) HCA of 38 key differential volatile compounds. S1, S2, S3 and S4 represented the moisture contents of 73.36%, 71.40%, 69.17%, and 67.53%, respectively.

To clarify the differences in volatile compounds of green tea across different spreading degrees, OPLS-DA was performed. As depicted in Figure 2A, the score plot revealed clear quadrant-specific clustering: S1 (first quadrant), S2 (fourth quadrant), S3 (second quadrant), and S4 (third quadrant). This spatial distribution pattern showed strong concordance with GC-E-Nose profiling results. The OPLS-DA model demonstrated strong interpretative and predictive performance, with R^2^Y = 0.957 and Q^2^ = 0.805. Additionally, permutation tests comprising 200 iterations were conducted to evaluate the model’s robustness. The obtained parameters (R^2^ = 0.737, Q^2^ = −0.607) confirmed that the model was reliable without evidence of overfitting (Figure 2B). Variable importance in projection (VIP) analysis identified 43 volatile compounds (VIP > 1.0) as discriminant variables for the classification. To illustrate how these key compounds influence group separation, their loadings are plotted in Figure 2C. In this plot, the coordinate of each variable reflects its correlation with the predictive component of the OPLS-DA model; variables positioned farther from the origin typically exert a stronger influence on the group separation visualized in the corresponding score plot. Based on this analysis, the characteristic volatile profile of each group was delineated as follows: the S1 group was associated with higher levels of eugenol (No. 57), *β*-ionone (No. 67), and indole (No. 52); the S2 group with *(E)*-2-hexenol (No. 8) and *β*-damascenone (No. 61); and the S3 group with *β*-cyclocitral (No. 43), safranal (No. 40), and butanoic acid, hexyl ester (No. 38). Notably, the S4 profile was distinguished by an enrichment of lipid oxidation-derived aldehydes, namely *(E*,*E)*-2,4-decadienal (No. 55), hexanal (No. 4), and *(E*,*E)*-2,4-heptadienal (No. 22), indicating an accentuation of lipoxygenase-mediated pathways under more intensive spreading conditions.

Subsequent dual-threshold filtering (VIP > 1, *p* < 0.05) refined this set to 38 key differential compounds, predominantly esters, aldehydes, and alcohols. HCA was conducted on 38 selected volatile compounds (Figure 2D). The color intensity gradients in the heatmap reflect the concentration variations across different spreading treatments. Specifically, red indicates concentrations above the average, with deeper red signifying greater abundance, whereas green represents concentrations below the average, with deeper green indicating a lower level. The HCA results corroborated the group-specific volatile patterns: compounds such as *cis*-jasmone, *β*-damascenone, *β*-ionone, nerolidol, and indole were enriched in S1; *(E)*-2-hexenol in S2; geranic acid and *β*-cyclocitral in S3; and hexanal, *(E*,*E)*-2,4-heptadienal and (*E*,*E*)-2,4-decadienal in S4. These distinct compositional patterns underpin the statistical separation of the samples, confirming that the identified volatiles are essential discriminators among the spreading treatments.

#### 3.3.3. OAV Analysis of Key Volatile Compounds

It is well-known that not every volatile compound found in green tea affects its aroma, with only a subset of them significantly influencing the overall aroma profile. Consequently, this study conducted an in-depth investigation of odor-active compounds through OAV analysis. Generally, volatiles with OAV ≥ 1 are deemed significant contributors to the characteristic aroma of green tea, with higher OAVs suggesting a more substantial effect on the overall aromatic profile.

**Figure 3:**
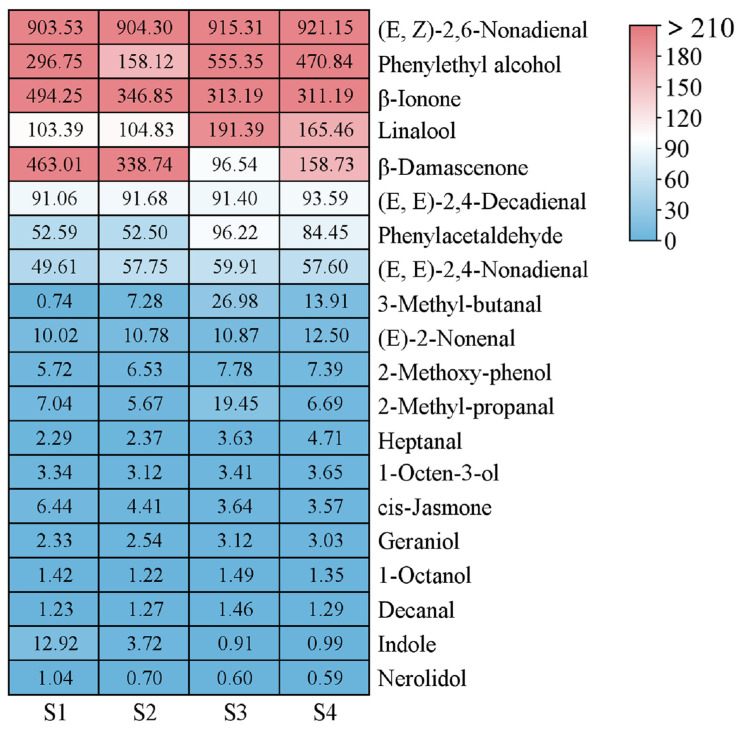
Comparison of OAVs of 21 key aroma compounds. S1, S2, S3 and S4 represented the moisture contents of 73.36%, 71.40%, 69.17%, and 67.53%, respectively.

In total, 21 volatile compounds exhibiting OAVs ≥ 1 were identified in tea samples that underwent four distinct levels of spreading (Figure 3). These key compounds primarily included 1-octanol, heptanal, 2-methoxy-phenol, phenylacetaldehyde, linalool, phenylethyl alcohol, *(E*,*Z)*-2,6-nonadienal, *(E)*-2-nonenal, decanal, *(E*,*E)*-2,4-nonadienal, geraniol, citral, indole, *(E*,*E)*-2,4-decadienal, *cis*-jasmone, *β*-damascenone, nerolidol, *β*-ionone, 3-methyl-butanal, 2-methyl-propanal, and 1-octen-3-ol. Among them, *(E*,*Z)*-2,6-nonadienal (OAV = 903.53~921.15), phenylethyl alcohol (OAV = 158.12~555.35), *β*-ionone (OAV = 311.19~494.25), linalool (OAV = 103.39~191.39), and *β-*damascenone (OAV = 96.54~463.01) exhibited higher OAVs, which substantially contribute to the unique aroma of green tea samples under different spreading degrees. The OAV data directly elucidate the sensory differences among treatments. The enhanced floral character of S1 corresponded to significantly higher OAVs of key floral odorants, particularly *β*-ionone and *β*-damascenone. Conversely, the shift toward greener, fatty, and malty notes in S3 and S4 was driven by elevated OAVs of lipid oxidation-derived aldehydes, such as *(E)*-2-nonenal and *(E*,*E)*-2,4-nonadienal.

#### 3.3.4. Analyze the Possible Pathways of Key Odorants

**Figure 4:**
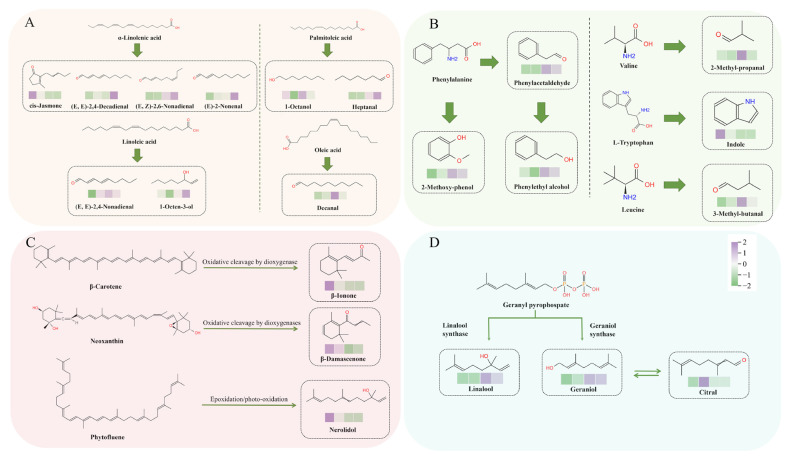
The possible formation pathways of 21 key odorants with OAVs > 1. (**A**) Fatty acid-derived volatiles; (**B**) Amino acid-derived volatiles; (**C**) Carotenoid-derived volatiles; (**D**) Glycoside-derived volatiles. The color blocks (left to right) represented samples S1–S4, with respective moisture contents of 73.36%, 71.40%, 69.17%, and 67.53%.

Based on distinct secondary metabolite biosynthesis pathways, the 21 key odorants were categorized into four distinct groups: 9 fatty acid-derived volatiles (FADVs) (Figure 4A), 6 amino acid-derived volatiles (AADVs) (Figure 4B), 3 carotenoid-derived volatiles (CDVs) (Figure 4C), and 3 glycoside-derived volatiles (GDVs) (Figure 4D) [22]. Among these, FADVs constituted the most predominant group, followed by AADVs, highlighting the essential roles of lipid degradation and amino acid degradation in the aroma formation across varying spreading treatments [23].

Specifically, a series of C6–C10 aliphatic FADVs, including *(E*,*E)*-2,4-decadienal, *cis*-jasmone, decanal, *(E)*-2-nonenal, 1-octanol, 1-octen-3-ol, heptanal, *(E*,*E)*-2,4-nonadienal, and *(E*,*Z)*-2,6-nonadienal, are generated from lipid precursors via lipoxygenase (LOX)-mediated oxidation [24,25]. During the spreading process, the reduction in leaf moisture content alters the intracellular microenvironment, leading to a gradual loss of cell turgor and increased membrane permeability. These changes promote the release and activation of endogenous enzymes such as LOX. Under mild spreading conditions, moderate water loss sustains enzymatic activity, thereby favoring the LOX-catalyzed oxidation of fatty acids such as α-linolenic acid and leading to the formation of key aroma volatiles like *(E*,*Z)*-2,6-nonadienal. These aldehydes can then be reduced to their corresponding alcohols by alcohol dehydrogenases (ADHs), contributing to the fresh and green aroma notes characteristic of tea. The specific precursor relationships are as follows: *cis*-jasmone, *(E*,*E)*-2,4-decadienal, *(E)*-2-nonenal, and *(E*,*Z)*-2,6-nonadienal are generated from α-linolenic acid, whereas *(E*,*E)*-2,4-nonadienal and 1-octen-3-ol originate from linolenic acid [26]. Additionally, 1-octanol and heptanal are produced from palmitoleic acid, and decanal is formed from oleic acid [27]. As spreading intensifies and moisture declines, the progression of oxidative and degradative reactions may alter the final profile of aldehydes and alcohols. In our study, compounds like decanal and *(E*,*E)*-2,4-nonadienal, associated with fatty and green aromas, showed relatively stable levels across treatments in our study, suggesting their formation may be less sensitive to spreading-induced changes in enzyme activity compared to other volatiles [28].

The AADVs comprised 2-methoxyphenol, phenylacetaldehyde, phenylethyl alcohol, indole, 3-methylbutanal, and 2-methylpropanal. These compounds are derived from the degradation of L-phenylalanine, tryptophan, leucine, and valine, respectively [29]. Among them, 3-methyl-butanal, 2-methoxy-phenol, phenylacetaldehyde, and phenylethyl alcohol were present at higher concentrations in samples S3 and S4 compared to other treatments (Figure 4B). This indicates that a higher spreading degree promotes the degradation of L-phenylalanine into these aromatic compounds, aligning with prior findings [30]. Conversely, indole, which imparts a characteristic aroma, was most abundant under the mild S1 treatment, indicating that lighter spreading may favor the conversion of tryptophan to indole [26].

*β*-Damascenone, *β*-ionone, and nerolidol are products resulting from the degradation of carotenoids. *β*-Ionone, characterized by a violet-like aroma, is generated through the thermal degradation of *β*-carotene. Its low odor threshold renders it a prominent component in the flavor profile of both green and black teas [31]. *β*-Damascenone is produced through the enzymatic oxidation of neoxanthin, resulting in the development of sweet and honey-like fragrances. Nerolidol, produced via the photooxidation of phytofluene, reached its highest concentration in treatment S1 [32]. Mild fermentation can promote the degradation of carotenoids, thereby driving the formation of floral aroma compounds.

For GDVs, geranyl pyrophosphate serves as the common biosynthetic precursor for linalool and geraniol, which are catalyzed by linalool synthase and geraniol synthase, respectively [33]. In fresh tea leaves, a substantial proportion of these monoterpenes exists as non-volatile, glycosidically bound forms. During spreading, endogenous glycosidases—such as *β*-primeverosidase and *β*-glucosidase—hydrolyze these glycosidic conjugates, releasing free volatile aglycons and contributing to the floral and fruity aroma notes [26]. As spreading progresses and leaf moisture declines, oxidative and degradative pathways become increasingly prominent. For instance, geraniol can be further oxidized to citral, consistent with the elevated citral levels observed in S2.

### 3.4. Identification of Key Volatile Compounds Influencing Floral Aroma Formation

**Figure 5:**
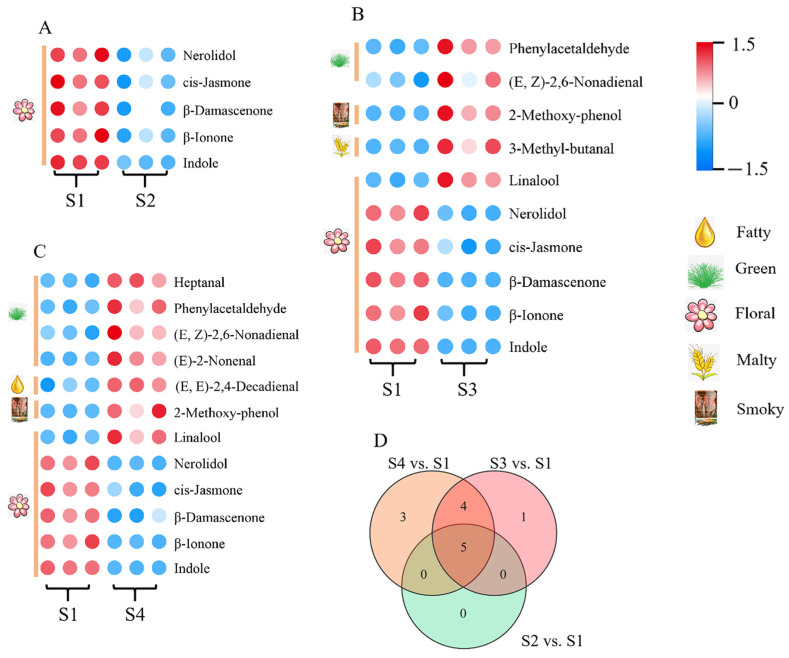
Key differential volatile compounds under various spreading degrees. (**A**) S2 vs. S1; (**B**) S3 vs. S1; (**C**) S4 vs. S1; (**D**) Venn diagram analysis. S1, S2, S3 and S4 represented the moisture contents of 73.36%, 71.40%, 69.17%, and 67.53%, respectively.

To directly bridge sensory perception with chemical composition, key volatile compounds influencing floral aroma formation were identified using a multi-criteria screening strategy (OAV > 1, VIP > 1, *p* < 0.05). This integrated approach prioritizes compounds with both high odor activity and strong discriminatory power among treatments. Comparative analyses were conducted between the highly floral aroma sample (S1) and three other groups: S2 (slightly floral aroma), S3 (non-floral aroma), and S4 (non-floral aroma). A total of 5, 10, and 12 differential volatile compounds were identified in the pairwise comparisons of S2 vs. S1, S3 vs. S1, and S4 vs. S1, respectively (Figure 5A–C). Specifically, S3 exhibited elevated levels of green-associated volatiles (phenylacetaldehyde, *(E*,*Z)*-2,6-nonadienal), malty (3-methylbutyral), and smoky (2-methoxyphenol) compounds relative to S1. Similarly, S4 exhibited increased concentrations of fatty ((*E*,*E*)-2,4-decadienal), green (heptanal, (*E*)-2-nonenal, phenylacetaldehyde, and (*E*,*Z*)-2,6-nonadienal), and smoky (2-methoxy-phenol) compounds. Notably, S1 exhibited significantly enriched floral markers, including nerolidol, *cis*-jasmone, *β*-damascenone, *β*-ionone, and indole, across all comparisons, aligning with established aroma-active signatures [34]. Venn analysis further identified five common floral volatiles present in all three comparisons: nerolidol, *cis*-jasmone, *β*-damascenone, *β*-ionone, and indole (Figure 5D). These compounds not only exhibited higher OAVs in S1 but also corresponded closely with the enhanced floral intensity scored by sensory evaluation, confirming their decisive roles as key drivers of floral aroma formation during spreading.

### 3.5. Interactions Between Key Floral Odorants and Olfactory Receptors via Molecular Docking

Odor perception is initiated by the activation of olfactory receptors through specific ligand-receptor interactions [35,36]. To explore the potential molecular interactions associated with floral aroma perception in green tea, molecular docking was performed between five key floral aroma compounds and a panel of ORs, including four broad-spectrum ORs (OR1A1, OR1D2, OR1G1, and OR2W1) and one narrow-spectrum OR (OR5M3). As shown in Table S6, the simulations demonstrated robust receptor-ligand interactions, with binding energies largely ranging below −5 kcal/mol, indicating thermodynamically favorable binding [37]. High-OAV compounds, particularly *β*-ionone and *β*-damascenone, exhibited stronger predicted affinities toward multiple ORs, suggesting a link between their high sensory impact and receptor-binding compatibility [38]. Notably, OR1D2 displayed the strongest binding affinity (<−6 kcal/mol) among all receptors, implicating its potential key role in floral odorant recognition. These findings align with established structure-activity relationships in olfactory signaling, wherein specific receptor conformations selectively recognize aroma-related ligands.

**Figure 6:**
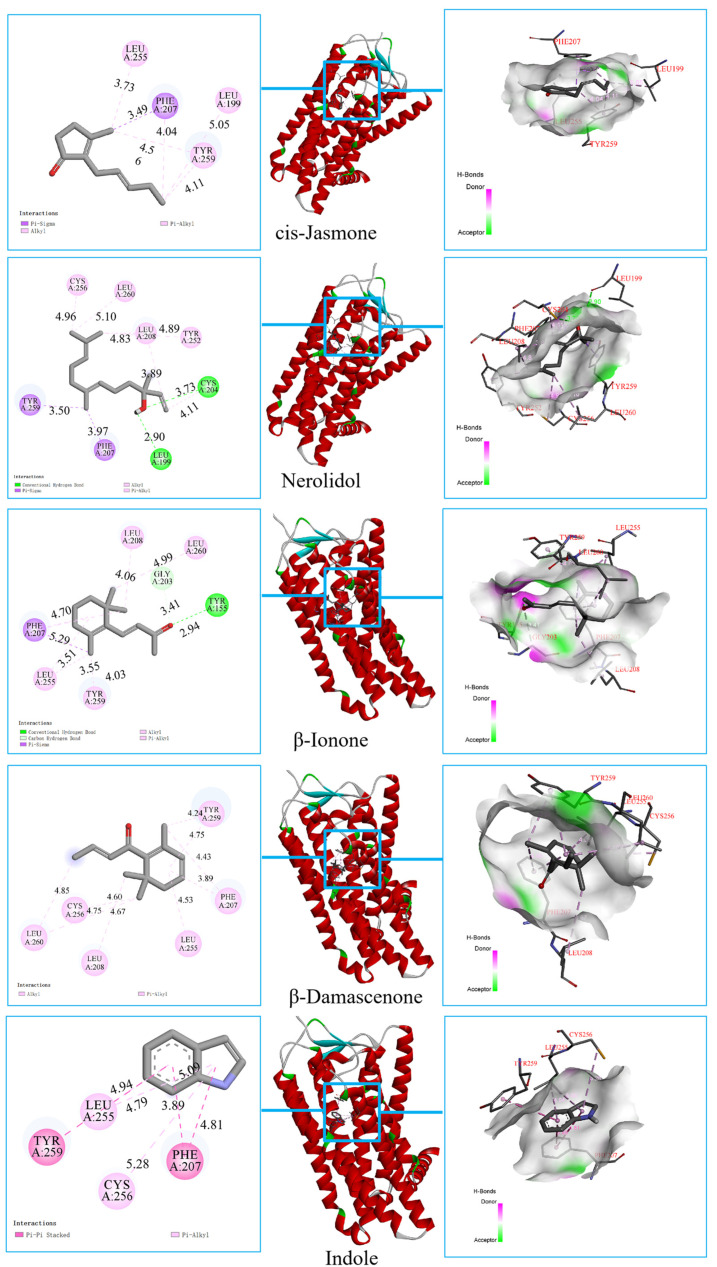
Molecular docking simulations of 5 key floral compounds with OR1D2.

Further analysis of the predicted interaction forces revealed potential molecular patterns underlying floral aroma perception (Figure 6 and Figures S3–S6). Hydrogen bonding consistently serves a significant function in the interactions between aromatic compounds and ORs [17]. Specific hydrogen-bond networks were identified between key floral compounds and receptor residues: for example, *β*-ionone with Gly203/Cys204 (OR1D2), Ser255 (OR1G1), and Asn176/Tyr178 (OR1A1); *cis*-jasmone with Thr (OR1D2); nerolidol with Leu199/Tyr155 (OR1A1); *β*-damascenone with Asn182 (OR1G1) and Ile254 (OR5M3); and indole with Asp180/Tyr278 (OR1G1) and Glu196 (OR2W1). In addition to hydrogen bonds, hydrophobic interactions with binding-site residues contributed substantially to ligand stabilization. Notably, even compounds with limited hydrogen-bonding capacity (e.g., indole with OR1A1, OR1D2, OR5M3) exhibited high binding energies, likely due to interactions with a cluster of hydrophobic residues that create a favorable nonpolar environment for stable binding [39]. Furthermore, multiple compounds were predicted to bind at identical receptor loci (e.g., Leu208, Phe207, Pro183, Tyr265), suggesting potential competitive binding dynamics among floral aroma constituents. Such spatial competition could lead to olfactory masking effects, wherein high-affinity compounds preferentially occupy shared receptor pockets and modulate overall perception [40,41].

## 4. Conclusions

This study employed the GC-E-Nose combined with targeted metabolomics to elucidate the mechanisms underlying the spreading degree-dependent modulation of aroma compounds of green tea. The findings indicated that mild spreading treatment (S1) developed superior aroma characteristics, particularly distinguished by its pronounced floral scent. Through GC-MS/MS-based quantitative profiling, 70 volatile compounds were systematically profiled, with ketones and heterocyclic compounds exhibiting progressive decrease as spreading intensity escalated as the spreading degrees increased. Multivariate analysis identified 38 differential compounds (VIP > 1.0, *p* < 0.05), among which *β*-ionone and *β*-damascenone displayed markedly enhanced OAVs in treatment S1, establishing their functional significance as floral note potentiators. By employing a tripartite screening strategy (OAV > 1, *p* < 0.05, VIP > 1), 5 pivotal volatile compounds (nerolidol, *cis*-jasmonone, *β*-damascenone, *β*-ionone, and indole) that influence the development of floral aroma characteristics in green tea were identified. Subsequent molecular docking analysis indicated that these five key floral compounds showed a stronger binding affinity for the olfactory receptor OR1D2. The analysis of binding modes indicated that hydrogen bonding and hydrophobic interactions were the primary molecular forces driving receptor-ligand binding. In addition, some key floral aroma compounds may produce mutual occlusion effects in the actual olfactory perception process by competitively binding to the same receptor site.

While these findings clarify the critical influence of spreading degree on aroma formation, it must be acknowledged that our research cannot definitively attribute whether volatile changes occur primarily during spreading or in subsequent fixation and drying. However, by integrating our results with established biochemistry, we posit that the spreading degree creates a foundational metabolic landscape—for instance, through enzyme activation and precursor liberation—which is later modified by subsequent thermal steps. These findings provide critical insights by identifying spreading degree as a key technological determinant for modulating floral aroma in green tea, thereby offering a scientific framework for precision control in tea manufacturing. To fully resolve the temporal dynamics of volatile formation, future studies should employ time-series sampling across all processing stages. Additionally, the molecular mechanisms of floral aroma perception warrant further investigation through techniques such as molecular dynamics simulations combined with electroencephalography.

## Data Availability

The original contributions presented in this study are included in the article/Supplementary Materials. Further inquiries can be directed to the corresponding authors.

## Supplementary Materials

The following supporting information can be downloaded at: https://www.mdpi.com/article/10.3390/foods15040735/s1. Figure S1: The processing processes of green tea samples subjected to different spreading degrees; Figure S2: The results of OPLS-DA obtained from GC-E-Nose. (A) The score plots of OPLS-DA (R^2^Y = 0.993, Q^2^ = 0.557); (B) Cross-validation by a 200-times permutation test (R^2^ = 0.745, Q^2^ = −0.609); Figure S3: Molecular docking simulation of 5 key floral compounds with OR1A1; Figure S4: Molecular docking simulation of 5 key floral compounds with OR1G1; Figure S5: Molecular docking simulation of 5 key floral compounds with OR1W1; Figure S6: Molecular docking simulation of 5 key floral compounds with OR5M3; Table S1: Standard information of volatile compounds used in this study; Table S2: The quantitative information of volatile compounds in tea samples under different spreading degrees; Table S3: The result of aroma quality of green tea based on sensory evaluation; Table S4: Characterization of the volatile compounds in tea samples across varying spreading degrees; Table S5: Threshold values and OAVs of volatile compounds in green tea across varying spreading degrees; Table S6: Summary of the binding energies between olfactory receptors and ligands. References [10,42,9,43,21] are cited in the Supplementary Materials.
