# Supplementary material for: Spreading Degree Modulates Floral Aroma Development in Green Tea: Integrated GC-E-Nose, Metabolomics, and Molecular Docking Reveals Key Odorants and Olfactory Receptor Interactions
Source: Foods. 2026 Feb 16;15(4):735. doi: 10.3390/foods15040735 (PMC12939911; doi:10.3390/foods15040735)
Supplement: Supplementary file 1 [file foods-15-00735-s001.zip › foods-4122907-supplementary.pdf]

Supporting Information for

Spreading Degree Modulates Floral Aroma Development in Green Tea: Integrated GC-E-Nose, Metabolomics, and Molecular Docking Reveals Key Odorants and Olfactory Receptor Interactions

Jiajing Hu [1,†], Xianxiu Zhou[1,2,†], Guangyue Hou[3], Jiahao Tang[1], Yongwen Jiang[1], Haibo Yuan[1], Daliang Shi[4,*] and Yanqin Yang[1,*]

[1]*National Key Laboratory for Tea Plant Germplasm Innovation and Resource Utilization, Tea Research Institute, Chinese Academy of Agricultural Sciences, Hangzhou 310008, China*

[2]*College of Tea Science, Yunnan Agricultural University, Kunming 650201, China*

[3]*Shandong Institute for Product Quality Inspection, Jinan 250102, China*

[4]*Tea Research Institute, Hangzhou Academy of Agriculture, Hangzhou 310024, China*

[*]*Correspondence: sdl7698@126.com (D.S.); yangyq@tricaas.com (Y.Y.)*

[†]*These authors contributed equally to this work.*

Table S1 Standard information of volatile compounds used in this study.

Standards	Related information			
	Purity	Brand	City	Country
Phenylethyl alcohol	99.50%	J&K	Shanghai	China
Linalool	98%	J&K	Shanghai	China
3-Methyl-butanal	99%	J&K	Shanghai	China
Decanal	97%	J&K	Shanghai	China
Butanoic acid, hexyl ester	>98%	J&K	Shanghai	China
Hexanoic acid, hexyl ester	99%	J&K	Shanghai	China
Phenylacetaldehyde	99%	Aladdin	Shanghai	China
Hexanoic acid, ethyl ester	>99%	Aladdin	Shanghai	China
Acetic acid, butyl ester	＞98%	Aladdin	Shanghai	China
1-Octen-3-ol	98%	Aladdin	Shanghai	China
2-Methyl-naphthalene	>97%	Aladdin	Shanghai	China
3-Carene	>90%	Aladdin	Shanghai	China
(*Z*)-3-Hexen-1-ol, acetate	98%	Aladdin	Shanghai	China
Nerol	>98%	Aladdin	Shanghai	China
Nerolidol	97%	Aladdin	Shanghai	China
2-Methoxy-phenol	98%	Aladdin	Shanghai	China
Hexanal	>98%	TCI	Shanghai	China
Heptanal	>98%	TCI	Shanghai	China
2-Methyl-propanal	>98%	TCI	Shanghai	China
1-Nonanal	>95%	TCI	Shanghai	China
β-Ionone	>95%	TCI	Shanghai	China
1-Hexanol	98%	TCI	Shanghai	China
Dihydroactinidiolide	≥99%	Medlife	Shanghai	China
α-Phellandrene	>95%	TCI	Shanghai	China
1-Penten-3-ol	98%	Macklin	Shanghai	China
Benzyl alcohol	99.50%	Macklin	Shanghai	China
Benzaldehyde	98%	Macklin	Shanghai	China
(*E*,*Z*)-2,6-Nonadienal	≥95%	Macklin	Shanghai	China
(*E*)-2-Nonenal	>95%	Macklin	Shanghai	China
(*E*,*E*)-2,4-Nonadienal	>90%	Macklin	Shanghai	China
(*E*,*E*)-2,4-Decadienal	>90%	Macklin	Shanghai	China
Methyl salicylate	>99%	Macklin	Shanghai	China
Hexanoic acid, methyl ester	99.50%	Macklin	Shanghai	China
Geranic acid	90%	Macklin	Shanghai	China
Eugenol	99%	Macklin	Shanghai	China
Benzoic acid, ethyl ester	>99.5%	Macklin	Shanghai	China
cis-Jasmone	98%	Macklin	Shanghai	China
3-Octanone	≥99.5%	Macklin	Shanghai	China
3-Nonen-2-one	>96%	Macklin	Shanghai	China
β-Damascenone	98%	Macklin	Shanghai	China

D-Limonene	99%	Macklin	Shanghai	China
Theaspirane	≥90%	Macklin	Shanghai	China
Butanoic acid, butyl ester	≥99.5%	Macklin	Shanghai	China
Propanoic acid, butyl ester	＞98%	Macklin	Shanghai	China
(*E*)-2-Hexenol	97%	Macklin	Shanghai	China
1,3,4,5-Tetramethylbenzene	＞70%	Macklin	Shanghai	China
Geranylacetone	97%	Meryer	Shanghai	China
Butanoic acid, 2-phenylethyl ester	98%	Meryer	Shanghai	China
(*E*)-2-Hexene-1-ol hexanoate	97%	Meryer	Shanghai	China
2,4-Dimethyl-1-heptene	≥98%	Meryer	Shanghai	China
1-Nonanol	99.50%	Yingxin	Shanghai	China
Citral	≥98%	Yingxin	Shanghai	China
Benzoic acid, 2-hydroxy-, ethyl ester	≥98%	Yingxin	Shanghai	China
(*E*)-2-Decenal	95%	Acmec	Shanghai	China
Butanoic acid, 2-methyl-, hexyl ester	98%	Acmec	Shanghai	China
1-Octanol	＞99.5%	Acmec	Shanghai	China
Geraniol	>98%	Yuanye	Shanghai	China
Butanoic acid, phenylmethyl ester	97.50%	Yuanye	Shanghai	China
(*E*)-2-Hexenal	≥99%	Yuanye	Shanghai	China
6-Methyl-5-hepten-2-one	98%	Alfa Aesar	Shanghai	China
Coumarin	≥99.5%	Vicbio	Beijing	China
cis-3-Hexenyl-α-methylbutyrate	96%	Heowns	Tianjin	China
β-Myrcene	98%	Naturewill	Sichuan	China
(*E*,*E*)-2,4-Heptadienal	≥90%	TRC	Toronto	Canada
Indole	＞97%	Solarbio	Beijing	China
Safranal	＞90%	Saitong	Beijing	China
δ-Cadinene	＞95%	D&B	Shanghai	China
α-Cyclocitral	98%	TRC	Toronto	Canada
β-Cyclocitral	95%	Macklin	Shanghai	China
(*E*)-2-Hexenyl butanoate	97%	Meryer	Shanghai	China

Table S2 The quantitative information of volatile compounds in tea samples under different spreading degrees.

No.	RT (min)	Compounds	Qualitative ion pairs	Quantitative ion pairs	Standard curves	R^2
1	1.92	2-Methyl-propanal	72→43	72→57	y=4304.98x+21697.81	0.993
2	2.55	3-Methyl-butanal	71→41	71→43	y=2449.46x+3364.93	0.995
3	2.98	1-Penten-3-ol	72→42	72→57	y=4167.53x+6616.27	0.994
4	6.02	Hexanal	72→44	72→43	y=43567.19x-52483.44	0.998
5	6.81	Acetic acid, butyl ester	56→54.2	56→41.2	y=225117.751x+48965.850	0.994
6	7.66	2,4-Dimethyl-1-heptene	126→70	126→83	y=22827.55x-11976.62	0.999
7	8.27	(*E*)-2-Hexenal	83→81.5	83→55	y=15836.338x+37526.248	0.997
8	8.79	(*E*)-2-Hexenol	91→61	91→65	y=3436.91x+12977.33	0.999
9	8.95	1-Hexanol	84→56	84→69	y=2141.12x-1072.38	0.999
10	10.36	Heptanal	70→41	70→55	y=98737.53x+297533.43	0.997
11	10.8	Propanoic acid, butyl ester	57.1→42	57.1→41	y=86096.769x+1442.054	0.999
12	11.58	Hexanoic acid, methyl ester	74→41	74→56.3	y=6046.527x+137.484	0.999
13	12.88	Benzaldehyde	77→74	77→50	y=2591.32x+1734.97	0.998
14	14.05	1-Octen-3-ol	99→71	99→43	y=10159.90x-4409.53	0.999
15	14.13	6-Methyl-5-hepten-2-one	108.1→93	108.1→79	y=1401.98x-84.95	0.998
16	14.15	3-Octanone	108.1→90.8	108.1→92.9	y=15763.45x+6793.69	0.993
17	14.31	*β*-Myrcene	93→77	93→91.2	y=90325.128x+5463.318	0.998
18	14.67	Butanoic acid, butyl ester	89→59	89→61.8	y=6741.602x-549.925	0.999
19	14.83	Hexanoic acid, ethyl ester	88→70.2	88→61	y=173031.921x-9315.301	0.999
20	14.94	*α*-Phellandrene	93.1→77	93.1→91	y=81896.337x+4966.399	0.998
21	15.1	3-Carene	93.1→77	93.1→91	y=9897.855x+10612.957	0.993
22	15.27	(*E,E*)-2,4-Heptadienal	110→95	110→81	y=47015.80x-145174.18	0.991
23	15.92	*D*-Limonene	93→77	93→91.2	y=124798.82x-92419.93	0.999

24	16.01	Benzyl alcohol	108→80	108→93	y=18161.41x-13410.60	0.999
25	16.4	Phenylacetaldehyde	120→117	120→105	y=3445.61x+14638.88	0.999
26	17.8	1-Octanol	84.1→69	84.1→56	y=40372.056x-2102.621	0.998
27	18.3	2-Methoxy-phenol	109→78	109→80	y=736.65x-3150.02	0.999
28	19.09	Linalool	93→77	93→91.2	y=27284.49x+123509.14	0.999
29	19.16	1-Nonanal	98→56	98→69	y=7247.74x-3304.40	0.999
30	19.53	Phenylethyl alcohol	122→97	122→92	y=3924.47x+2096.28	0.999
31	20.36	3-Nonen-2-one	125→92	125→94	y=22.91x+10.72	0.996
32	20.72	1,2,3,5-Tetramethylbenzene	134→108	134→89.8	y=47.878x-22.985	0.995
33	21.06	(*E,Z*)-2,6-Nonadienal	70→55	70→42	y=71904.86x-283393.69	0.998
34	21.43	(*E*)-2-Nonenal	83→41	83→55	y=94344.73x-335643.29	0.997
35	21.62	Benzoic acid, ethyl ester	122→109	122→120	y=1664.087x+3572.989	0.996
36	22.18	1-Nonanol	97→69	97→55	y=93780.72x-293297.69	0.995
37	22.51	Methyl salicylate	152→136	152→121	y=4984.36x-18082.07	0.999
38	22.54	Butanoic acid, hexyl ester	89.1→58.5	89.1→43	y=33852.109x-20168.163	0.999
39	22.77	(*E*)-2-Hexenyl butanoate	71.1→41	71.1→43	y=217866.566x+335708.510	0.99
40	22.8	Safranal	107.1→79	107.1→90.8	y=7029.397x+361.719	0.997
41	23.15	Decanal	112→69	112→70	y=38075.74x-89821.97	0.994
42	23.2	*cis*-3-Hexenyl acetate	89→84.3	89→63.3	y=336.825x-25.243	0.999
43	23.47	*β*-Cyclocitral	152.1→49	152.1→96	y=639.632x+2437.005	0.992
44	23.52	(*E,E*)-2,4-Nonadienal	81→79	81→53	y=6069.34x-17269.68	0.996
45	23.89	*cis*-3-Hexenyl-α-methylbutyrate	82.1→54	82.1→66.8	y=333191.283x-5847.491	0.999
46	24.05	Nerol	93→77	93→91	y=2660.503x+420.402	0.991
47	24.11	Butanoic acid, 2-methyl-, hexyl ester	85→43.2	85→57	y=280218.753x+6945.230	0.999
48	24.78	Geraniol	93→77	93→91	y=11028.11x-41301.85	0.998
49	24.8	(*E*)-2-Decenal	70→42.2	70→55	y=112723.849x-62877.371	0.999

50	25.3	Citral	84→41	84→56	y=11094.96x-30499.48	0.995
51	25.34	Benzoic acid, 2-hydroxy-, ethyl ester	120.1→88	120.1→92	y=114663.174x-174621.204	0.998
52	25.7	Indole	117→115	117→90	y=3299.50x-15025.98	0.999
53	26.01	Theaspirane	138→109	138→96	y=201627.62x-143935.19	0.998
54	26.43	2-Methyl-naphthalene	115→96.6	115→73	y=115.964x-263.021	0.998
55	27.31	(*E,E*)-2,4-Decadienal	81→79	81→53	y=13670.45x-32470.06	0.994
56	28.05	Butanoic acid, phenylmethyl ester	108.1→66	108.1→78.8	y=121.554x-233.346	0.998
57	28.64	Eugenol	149→125	149→119	y=441.331x+362.413	0.999
58	28.75	Geranic acid	100→79.8	100→81.7	y=442.639x-1075.339	0.993
59	29.2	(*Z*)-Hexanoic acid, 3-hexenyl ester	99→57	99→71	y=87922.59x-8886.50	0.996
60	29.41	Hexanoic acid, hexyl ester	117.1→90.8	117.1→86	y=904.385x-291.118	0.997
61	29.57	*β*-Damascenone	123→89	123→95	y=10991.77x-5539.93	0.999
62	29.7	*cis*-Jasmone	164→150	164→135	y=3667.95x-8130.43	0.999
63	29.76	(*E*)-2-Hexene-1-ol hexanoate	99.1→62	99.1→82.3	y=29.849x-16.387	0.994
64	30.52	Coumarin	146→135.4	146→140.7	y=20.2429x-47.950	0.994
65	31.14	Butanoic acid, 2-phenylethyl ester	104.1→84.3	104.1→80.4	y=20.182x-6.857	0.999
66	31.4	Geranyl acetone	136→108	136→121	y=34497.13x-58944.40	0.995
67	32.3	*β*-Ionone	177→107	177→121	y=976.25x-5819.20	0.994
68	33.48	*δ*-Cadinene	161.2→131	161.2→105.1	y=36443.296x-59.783	0.999
69	33.85	Nerolidol	93→77	93→91	y=27713.67x-146729.74	0.997
70	25.65	Dihydroactinidiolide	111→96	117→55	y=33356.88x-934.78	0.998

Table S3 The result of aroma quality of green tea based on sensory evaluation.

Samples	S1	S2	S3	S4
Comments	Floral	Slightly floral	Pure, full-bodied	Pure, full-bodied
Scores	90.83±0.29[a]	89.00±1.00[b]	87.17±0.42[c]	85.77±0.54[d]

Note: S1, S2, S3 and S4 represented the moisture contents of 73.36%, 71.40%, 69.17%, and 67.53%, respectively. Different letters indicated a significant difference ($p < 0.05$).

Table S4 Characterization of the volatile compounds in tea samples across varying spreading degrees.

No.	Compounds	Categories	Mean content ± standard deviation (μg/L)				
			S4	S3	S2	S1	*P*-values
1	2-Methyl-propanal	Aldehydes	3.28±5.67	9.53±7.86	2.78±4.81	3.45±4.97	0.498
2	3-Methyl-butanal	Aldehydes	15.30±19.18	29.68±8.25	8.01±10.63	0.81±0.56	0.078
3	1-Penten-3-ol	Alcohols	16.51±21.62	31.71±10.77	6.81±9.57	1.43±0.36	0.087
4	Hexanal	Aldehydes	1.36±0.05	1.28±0.04	1.30±0.01	1.28±0.01	0.029
5	Acetic acid, butyl ester	Esters	2.32±0.39	2.31±0.34	1.26±0.10	1.29±0.06	0.001
6	2,4-Dimethyl-1-heptene	Alkenes	0.69±0.11	0.57±0.01	0.56±0.01	0.57±0.02	0.053
7	(*E*)-2-Hexenal	Aldehydes	0.11±0.19	1.52±2.63	0.00±0.00	0.00±0.00	0.461
8	(*E*)-2-Hexenol	Alcohols	1.65±1.28	2.06±0.32	23.07±10.21	1.39±0.70	0.002
9	1-Hexanol	Alcohols	20.61±1.44	18.39±1.73	11.73±0.29	10.23±0.84	<0.001
10	Heptanal	Aldehydes	4.24±0.30	3.27±0.79	2.13±0.10	2.06±0.15	0.001
11	Propanoic acid, butyl ester	Esters	0.38±0.03	0.49±0.08	0.13±0.21	0.01±0.01	0.003
12	Hexanoic acid, methyl ester	Esters	0.02±0.02	0.03±0.05	0.02±0.03	0.02±0.04	0.966
13	Benzaldehyde	Aldehydes	8.54±0.34	7.01±0.92	4.55±0.73	3.53±0.19	<0.001
14	1-Octen-3-ol	Alcohols	3.65±0.32	3.41±0.55	3.12±0.11	3.34±0.42	0.444

15	6-Methyl-5-hepten-2-one	Ketones	0.28±0.08	0.78±0.79	0.78±0.95	1.19±1.21	0.653
16	3-Octanone	Ketones	0.15±0.14	0.01±0.02	0.00±0.00	0.03±0.06	0.147
17	*β*-Myrcene	Terpenes	1.87±0.36	2.00±0.19	1.21±0.08	1.45±0.07	0.006
18	Butanoic acid, butyl ester	Esters	0.09±0.00	0.12±0.00	0.12±0.00	0.12±0.00	<0.001
19	Hexanoic acid, ethyl ester	Esters	0.07±0.00	0.09±0.00	0.09±0.00	0.09±0.00	<0.001
20	*α*-Phellandrene	Terpenes	1.87±0.35	2.01±0.16	1.20±0.10	1.45±0.07	0.004
21	3-Carene	Alkenes	30.17±7.98	32.96±4.67	18.02±0.89	37.11±8.84	0.032
22	(*E,E*)-2,4-Heptadienal	Aldehydes	4.76±0.19	4.20±0.16	4.22±0.12	4.11±0.26	0.01
23	*D*-Limonene	Terpenes	1.31±0.21	1.19±0.06	1.05±0.06	1.23±0.08	0.145
24	Benzyl alcohol	Alcohols	1.16±0.17	1.05±0.05	0.97±0.04	1.09±0.06	0.186
25	Phenylacetaldehyde	Aldehydes	101.34±9.38	115.46±12.74	63.00±7.81	63.11±3.62	<0.001
26	1-Octanol	Alcohols	4.05±0.68	4.48±0.58	3.66±0.37	4.27±0.03	0.27
27	2-Methoxy-phenol	Phenols	6.21±0.41	6.53±0.43	5.49±0.60	4.81±0.03	0.004
28	Linalool	Alcohols	99.27±9.08	114.83±12.09	62.90±8.04	62.04±3.61	<0.001
29	1-Nonanal	Aldehydes	1.18±0.48	1.33±0.19	1.14±0.23	0.95±0.13	0.491
30	Phenylethyl alcohol	Alcohols	164.79±79.51	194.37±53.21	55.34±39.78	103.86±15.89	0.046
31	3-Nonen-2-one	Ketones	0.66±0.36	0.99±0.59	0.62±0.18	0.50±0.57	0.622
32	1,2,3,5-Tetramethylbenzene	Aromatic hydrocarbons	0.49±0.43	0.74±0.21	0.62±0.10	0.77±0.21	0.58
33	(*E*,*Z*)-2,6-Nonadienal	Aldehydes	4.15±0.03	4.12±0.02	4.07±0.02	4.07±0.02	0.009
34	(*E*)-2-Nonenal	Aldehydes	5.00±0.20	4.35±0.46	4.31±0.21	4.01±0.04	0.012
35	Benzoic acid, ethyl ester	Esters	22.05±3.76	33.92±7.48	14.07±1.46	15.90±1.47	0.002
36	1-Nonanol	Alcohols	3.94±0.01	3.74±0.05	3.65±0.05	3.65±0.02	<0.001
37	Methyl salicylate	Esters	37.25±7.58	36.31±5.04	21.16±1.80	19.59±0.99	0.002
38	Butanoic acid, hexyl ester	Esters	0.85±0.04	1.12±0.03	0.76±0.01	0.80±0.05	<0.001
39	(*E*)-2-Hexenyl butanoate	Esters	0.07±0.08	1.08±0.49	0.37±0.31	2.23±1.00	0.008
40	Safranal	Aldehydes	1.92±0.10	2.62±0.04	1.87±0.29	2.14±0.08	0.002

41	Decanal	Aldehydes	3.36±0.11	3.80±0.26	3.29±0.10	3.19±0.33	0.044
42	*cis*-3-Hexenyl acetate	Esters	0.26±0.14	0.50±0.53	0.33±0.18	0.40±0.11	0.78
43	*β*-Cyclocitral	Aldehydes	0.00±0.00	0.85±0.11	0.00±0.00	0.09±0.08	<0.001
44	(*E*,*E*)-2,4-Nonadienal	Aldehydes	3.46±0.50	3.59±0.94	3.47±0.86	2.98±0.04	0.707
45	*cis*-3-Hexenyl-α-methylbutyrate	Esters	3.09±0.17	6.50±0.70	3.92±0.08	5.31±0.40	<0.001
46	Nerol	Alcohols	60.44±9.13	69.57±7.80	51.98±6.44	42.09±1.69	0.007
47	Butanoic acid, 2-methyl-, hexyl ester	Esters	1.71±0.37	3.46±0.32	1.06±0.06	0.88±0.07	<0.001
48	Geraniol	Alcohols	22.75±6.98	23.38±3.70	19.05±6.78	17.51±0.95	0.487
49	(*E*)-2-Decenal	Aldehydes	0.93±0.32	0.87±0.15	0.81±0.04	0.81±0.04	0.803
50	Citral	Aldehydes	3.93±0.29	3.92±0.26	5.97±4.69	3.33±0.02	0.565
51	Benzoic acid, 2-hydroxy-, ethyl ester	Esters	1.53±0.00	1.54±0.00	1.53±0.00	1.53±0.00	0.007
52	Indole	Heterocyclic compounds	10.91±1.78	10.05±0.73	40.92±5.20	142.09±4.20	<0.001
53	Theaspirane	Heterocyclic compounds	0.81±0.02	0.83±0.02	0.81±0.01	0.82±0.02	0.393
54	2-Methyl-naphthalene	Aromatic hydrocarbons	4.15±0.12	7.23±0.63	5.18±0.10	6.98±0.21	<0.001
55	(*E*,*E*)-2,4-Decadienal	Aldehydes	2.53±0.01	2.47±0.01	2.48±0.03	2.46±0.02	0.008
56	Butanoic acid, phenylmethyl ester	Esters	18.87±3.65	22.48±1.80	19.06±0.50	22.26±1.51	0.14
57	Eugenol	Phenols	0.49±0.28	0.66±0.16	1.23±0.05	3.02±0.32	<0.001
58	Geranic acid	Acids	4.30±1.05	6.59±1.30	4.29±0.46	5.58±0.61	0.041
59	(*Z*)-Hexanoic acid, 3-hexenyl ester	Esters	16.01±5.75	35.42±7.24	30.90±3.75	35.99±1.35	0.004
60	Hexanoic acid, hexyl ester	Esters	0.98±0.19	2.20±0.20	1.00±0.06	1.59±0.23	<0.001
61	*β*-Damascenone	Ketones	0.95±0.60	0.58±0.02	2.03±0.30	2.78±0.15	<0.001
62	cis-Jasmone	Ketones	25.02±4.24	25.51±5.43	30.88±4.10	45.11±2.44	0.001
63	(*E*)-2-Hexene-1-ol hexanoate	Esters	1.48±0.43	1.48±0.49	1.10±0.09	2.06±1.15	0.418
64	Coumarin	Esters	1.73±1.50	2.64±0.20	3.34±0.79	6.14±2.77	0.05
65	Butanoic acid, 2-phenylethyl ester	Esters	0.31±0.27	0.49±0.08	1.21±0.65	0.55±0.27	0.083
66	Geranyl acetone	Ketones	3.04±0.10	3.17±0.33	3.00±0.11	3.16±0.13	0.635

67	*β*-Ionone	Ketones	6.54±0.11	6.58±0.32	7.28±0.71	10.38±0.50	<0.001
68	*δ*-Cadinene	Terpenes	0.16±0.09	0.16±0.03	0.20±0.11	0.24±0.08	0.593
69	Nerolidol	Alcohols	5.92±0.12	6.01±0.33	7.01±0.89	10.40±0.51	<0.001
70	Dihydroactinidiolide	Esters	0.50±0.24	0.36±0.05	0.48±0.24	0.31±0.00	0.49

Note: S1, S2, S3 and S4 represented the moisture contents of 73.36%, 71.40%, 69.17%, and 67.53%, respectively.

Table S5 Threshold values and OAVs of volatile compounds in green tea across varying spreading degrees.

			OAVs			
No.	Compounds	Odor thresholds (μg/L)	S4	S3	S2	S1
1	2-Methyl-propanal*	0.49 [A]	6.69	19.45	5.67	7.04
2	3-Methyl-butanal*	1.1 [B]	13.91	26.98	7.28	0.74
3	1-Penten-3-ol	400 [C]	0.04	0.08	0.02	0
4	Hexanal	4.5 [C]	0.3	0.29	0.29	0.28
5	Acetic acid, butyl ester	100 [B]	0.02	0.02	0.01	0.01
6	2,4-Dimethyl-1-heptene	n.f.	/	/	/	/
7	(*E*)-2-Hexenal	17 [C]	0.01	0.09	0	0
8	(*E*)-2-Hexenol	232 [C]	0.01	0.01	0.1	0.01
9	1-Hexanol	500 [C]	0.04	0.04	0.02	0.02
10	Heptanal*	0.9 [C]	4.71	3.63	2.37	2.29
11	Propanoic acid, butyl ester	200 [C]	0	0	0	0
12	Hexanoic acid, methyl ester	70 [C]	0	0	0	0
13	Benzaldehyde	350 [C]	0.02	0.02	0.01	0.01
14	1-Octen-3-ol*	1 [C]	3.65	3.41	3.12	3.34
15	6-Methyl-5-hepten-2-one	50 [C]	0.01	0.02	0.02	0.02

16	3-Octanone	21.4 [B]	0.01	0	0	0
17	*β*-Myrcene	15 [C]	0.12	0.13	0.08	0.1
18	Butanoic acid, butyl ester	100 [B]	0	0	0	0
19	Hexanoic acid, ethyl ester	5 [C]	0.01	0.02	0.02	0.02
20	*α*-Phellandrene	160 [C]	0.01	0.01	0.01	0.01
21	3-Carene	4000 [C]	0.01	0.01	0	0.01
22	(*E,E*)-2,4-Heptadienal	10000 [C]	0	0	0	0
23	*D*-Limonene	34 [C]	0.04	0.04	0.03	0.04
24	Benzyl alcohol	100 [C]	0.01	0.01	0.01	0.01
25	Phenylacetaldehyde*	1.2 [C]	84.45	96.22	52.5	52.59
26	1-Octanol*	3 [B]	1.35	1.49	1.22	1.42
27	2-Methoxy-phenol*	0.84 [A]	7.39	7.78	6.53	5.72
28	Linalool*	0.6 [C]	165.46	191.39	104.83	103.39
29	1-Nonanal	2.8 [A]	0.42	0.47	0.41	0.34
30	Phenylethyl alcohol*	0.35 [C]	470.84	555.35	158.12	296.75
31	3-Nonen-2-one	800 [B]	0	0	0	0
32	1,2,3,5-Tetramethylbenzene	n.f.	/	/	/	/
33	(*E,Z*)-2,6-Nonadienal*	0.0045 [A]	921.15	915.31	904.3	903.53
34	(*E*)-2-Nonenal*	0.4 [A]	12.5	10.87	10.78	10.02
35	Benzoic acid, ethyl ester	56 [C]	0.39	0.61	0.25	0.28
36	1-Nonanol	45.5 [C]	0.09	0.08	0.08	0.08
37	Methyl salicylate	40 [C]	0.93	0.91	0.53	0.49
38	Butanoic acid, hexyl ester	250 [C]	0	0	0	0
39	(*E*)-2-hexenyl butanoate	3130 [E]	0	0	0	0
40	Safranal	3 [D]	0.64	0.87	0.62	0.71
41	Decanal*	2.6 [C]	1.29	1.46	1.27	1.23

42	*cis*-3-Hexenyl acetate	n.f.	/	/	/	/
43	*β*-Cyclocitral	3 [E]	0	0.17	0	0.02
44	(*E*,*E*)-2,4-Nonadienal*	0.06 [A]	57.6	59.91	57.75	49.61
45	*cis*-3-Hexenyl-α-methylbutyrate	n.f.	/	/	/	/
46	Nerol	290 [C]	0.21	0.24	0.18	0.15
47	Butanoic acid, 2-methyl-, hexyl ester	22 [C]	0.08	0.16	0.05	0.04
48	Geraniol*	7.5 [C]	3.03	3.12	2.54	2.33
49	(*E*)-2-Decenal	2.7 [C]	0.34	0.32	0.3	0.3
50	Citral*	5 [A]	0.79	0.78	1.19	0.67
51	Benzoic acid, 2-hydroxy-, ethyl ester	84 [C]	0.02	0.02	0.02	0.02
52	Indole*	11 [A]	0.99	0.91	3.72	12.92
53	Theaspirane	n.f.	/	/	/	/
54	2-Methyl-naphthalene	10 [C]	0.41	0.72	0.52	0.7
55	(*E,E*)-2,4-decadienal*	0.027 [A]	93.59	91.4	91.68	91.06
56	Butanoic acid, phenylmethyl ester	376 [C]	0.05	0.06	0.05	0.06
57	Eugenol	150 [C]	0	0	0.01	0.02
58	Geranic acid	n.f.	/	/	/	/
59	(*Z*)-Hexanoic acid, 3-hexenyl ester	781 [D]	0.02	0.05	0.04	0.05
60	Hexanoic acid, hexyl ester	6400 [C]	0	0	0	0
61	*β*-Damascenone*	0.006 [A]	158.73	96.54	338.74	463.01
62	*cis*-Jasmone*	7 [C]	3.57	3.64	4.41	6.44
63	(*E*)-2-Hexene-1-ol hexanoate	195 [C]	0.01	0.01	0.01	0.01
64	Coumarin	11 [C]	0.16	0.24	0.3	0.56
65	Butanoic acid, 2-phenylethyl ester	376 [C]	0	0	0	0
66	Geranyl acetone	60 [C]	0.05	0.05	0.05	0.05
67	*β*-Ionone*	0.021 [D]	311.19	313.19	346.85	494.25

68	δ-Cadinene	1.5 [C]	0.11	0.11	0.13	0.16
69	Nerolidol*	10 [C]	0.59	0.6	0.7	1.04
70	Dihydroactinidiolide	500 [B]	0	0	0	0

Note: OAV represented odor activity value. * represented compounds with OAV >1 in the four treatments; S1, S2, S3 and S4 represented the moisture contents of 73.36%, 71.40%, 69.17%, and 67.53%, respectively. The odor thresholds of volatile components cited in this file are detailed in the main text (A, B, C, D, and E correspond to references 10, 42, 9, 43, and 21, respectively).

Table S6 Summary of the binding energies between olfactory receptors and ligands.

Ligands	Binding energy (kcal/mol)				
	OR1A1	OR1D2	OR1G1	OR2W1	OR5M3
cis-Jasmone	-6.4	-6.8	-5.4	-5.2	-6.2
Nerolidol	-5.8	-7	-6	-6.2	-6.4
β-Damascenone	-6.3	-7.6	-6.2	-6.1	-6.9
β-Ionone	-5.8	-8.2	-6.1	-6.1	-6.9
Indole	-6.3	-6.7	-5	-5.5	-5.9

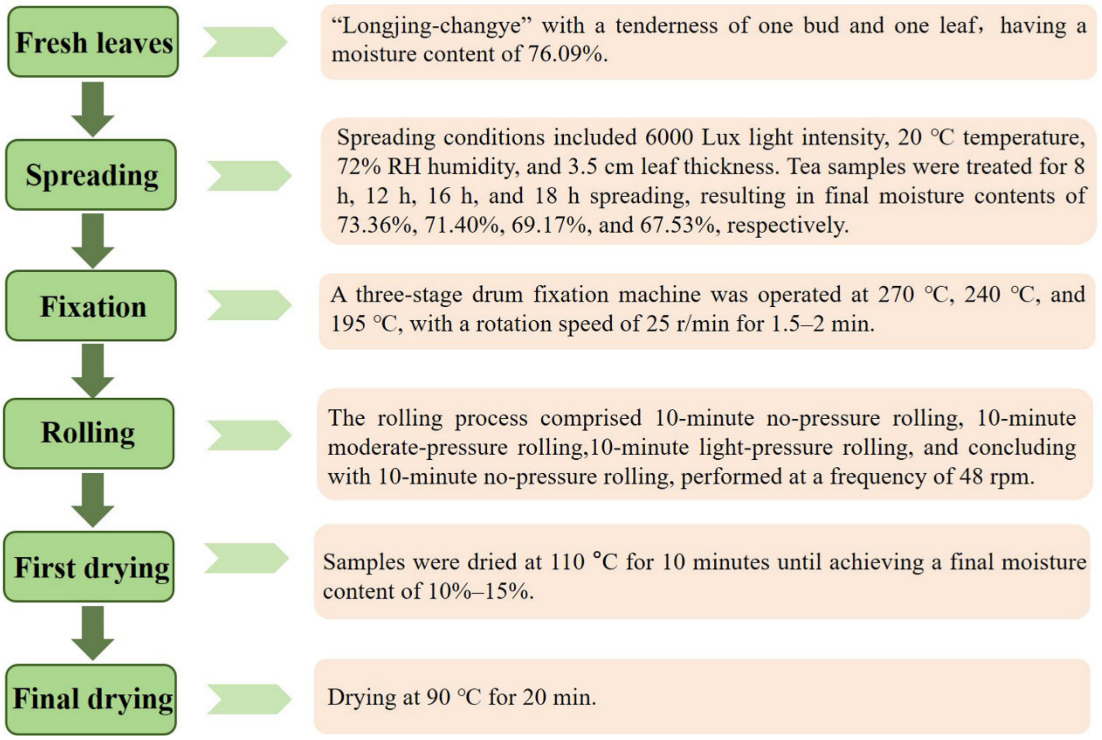

Figure S1 The processing processes of green tea samples subjected to different spreading degrees.

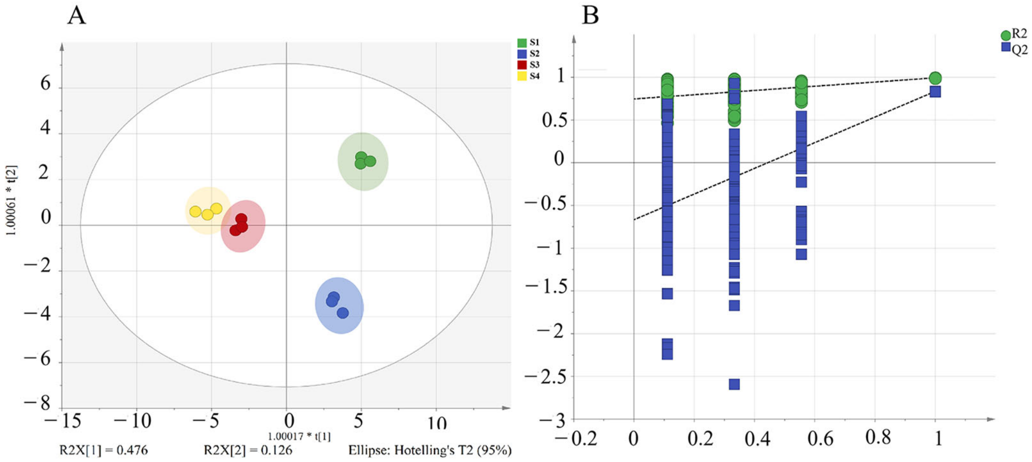

Figure S2 The results of OPLS-DA obtained from GC-E-Nose. (A) The score plots of OPLS-DA ($R^2Y = 0.993$, $Q^2 = 0.557$); (B) Cross-validation by a 200-times permutation test ($R^2 = 0.745$, $Q^2 = -0.609$).

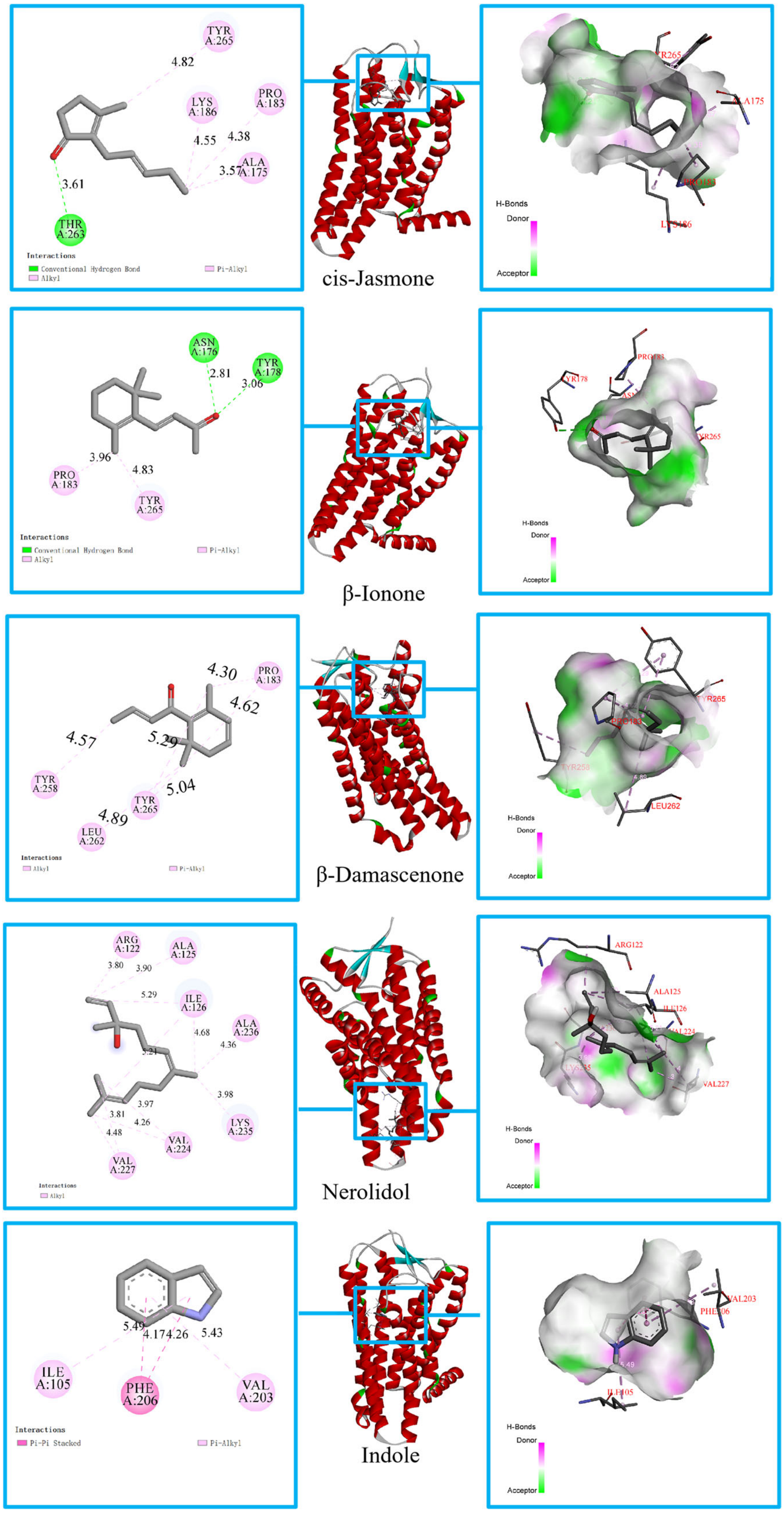

Figure S3 Molecular docking simulation of 5 key floral compounds with OR1A1.

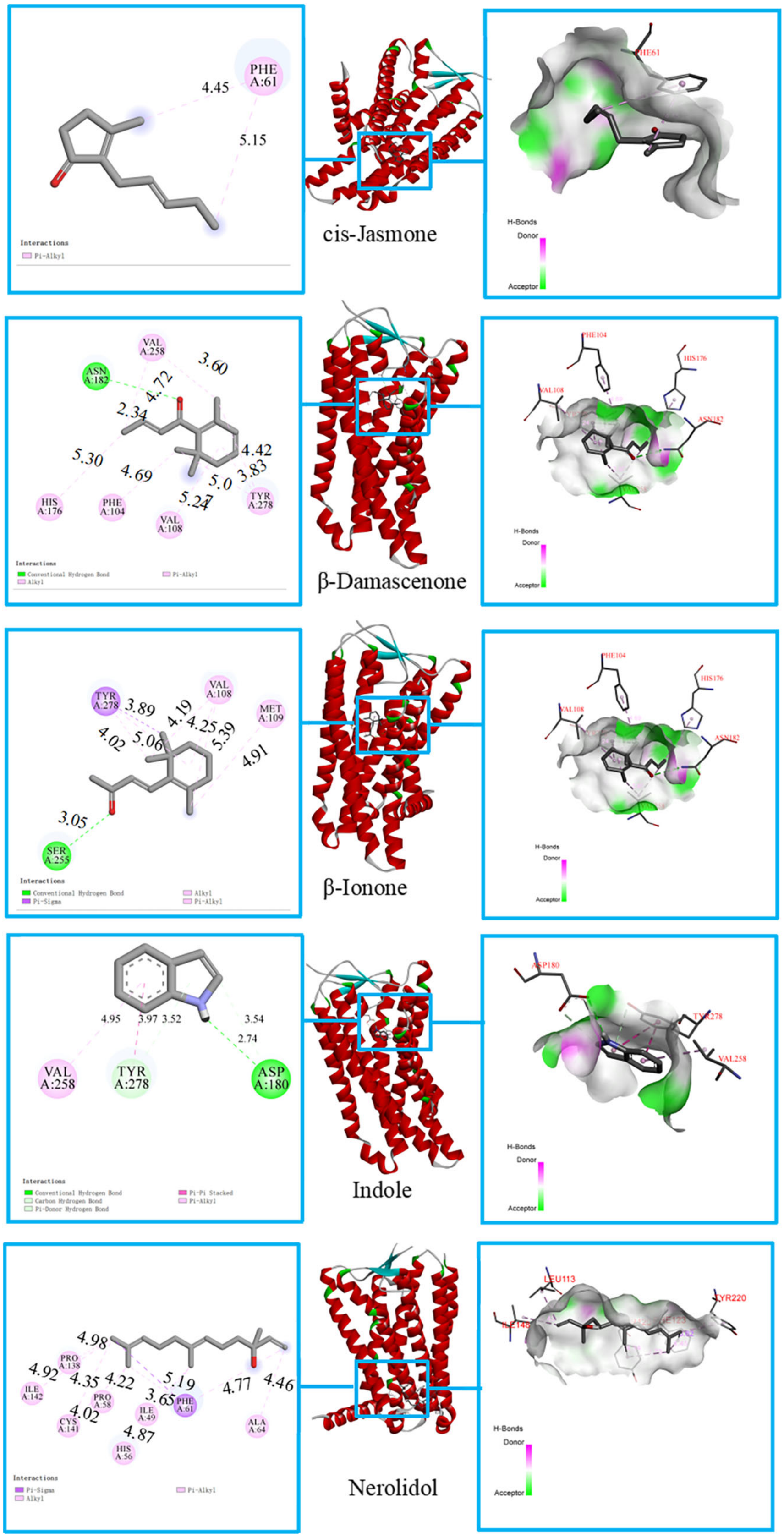

Figure S4 Molecular docking simulation of 5 key floral compounds with OR1G1.

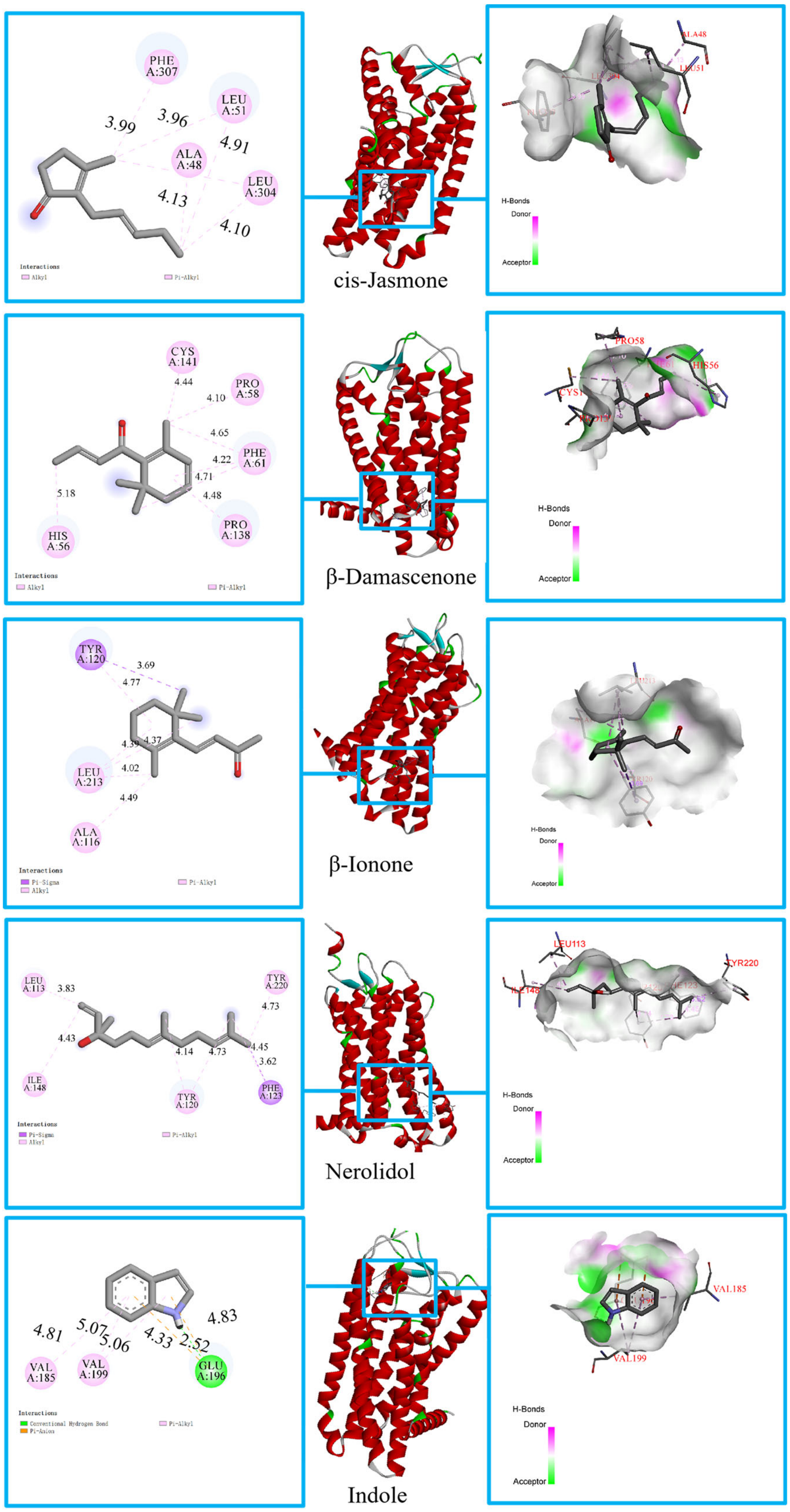

Figure S5 Molecular docking simulation of 5 key floral compounds with OR1W1.

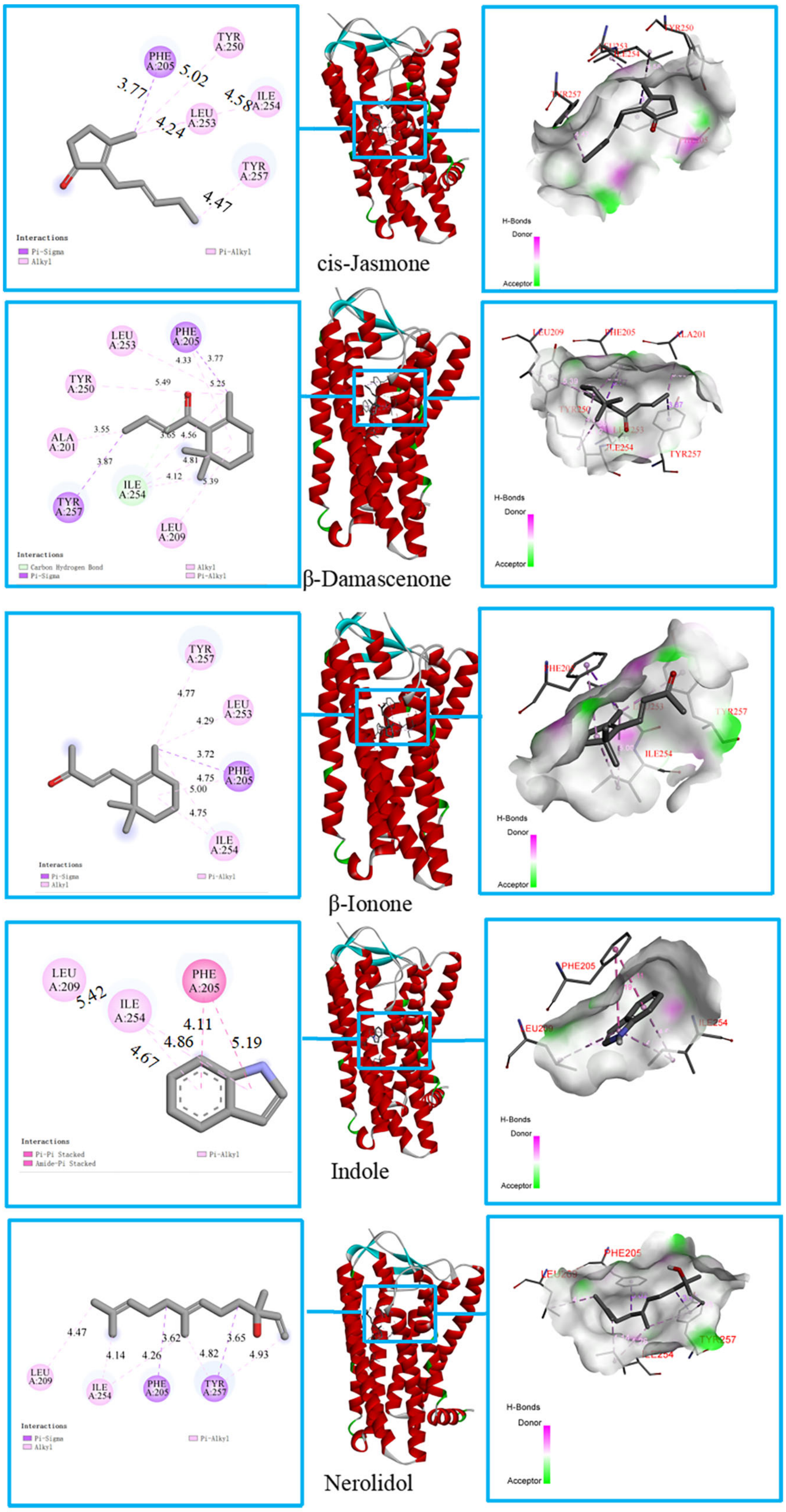

Figure S6 Molecular docking simulation of 5 key floral compounds with OR5M3.
